# A mature T-cell neoplasm with features of peripheral T-cell lymphoma, not otherwise specified presenting with multiple cutaneous tumors on the scalp in a patient with long-standing lichen planopilaris

**DOI:** 10.1016/j.jdcr.2025.01.037

**Published:** 2025-03-04

**Authors:** Brittani A. Jones, Wesley T. Hodges, Mahta Salehi, Georgeanne Cornell, Sagun Goyal, John L. Frater, M. Yadira Hurley

**Affiliations:** a Department of Dermatology, SLUCare Physician Group, Saint Louis, Missouri; b Department of Hematology and Oncology, SLUCare Physician Group, Saint Louis, Missouri; c Department of Pathology and Immunology, Washington University, Saint Louis, Missouri

**Keywords:** lichen planopilaris, LPP, peripheral T-cell lymphoma, PTCL, PTCL-NOS

## Introduction

Mature T-cell neoplasms (MTCNs) are diverse, with highly variable clinical presentation, natural history, and outcomes.^1^ Peripheral T-cell lymphoma, not otherwise specified (PTCL-NOS) is the most commonly seen subtype, comprising ∼25% of mature T-cell neoplasms and ∼10 to 12% of all lymphoid malignancies, and is encountered in lymph nodes and extranodal sites, including the skin.^2^^,^^3^

Lymphocytic infiltration of the skin can also be seen in benign inflammatory conditions. Lichen planopilaris (LPP), considered a variant of lichen planus, is a scarring alopecia characterized histologically by dense perifollicular infiltration of lymphocytes along the upper third of the hair follicle. LPP has been associated with other inflammatory conditions such as autoimmune thyroid diseases,^4^ but to the best of our knowledge, no association has been established between LPP and the development of leukemias/lymphomas. Herein, we report a case of MTCN with features of PTCL-NOS presenting with multiple cutaneous tumors in a patient with long-standing LPP.

## Case

**Fig 1:**
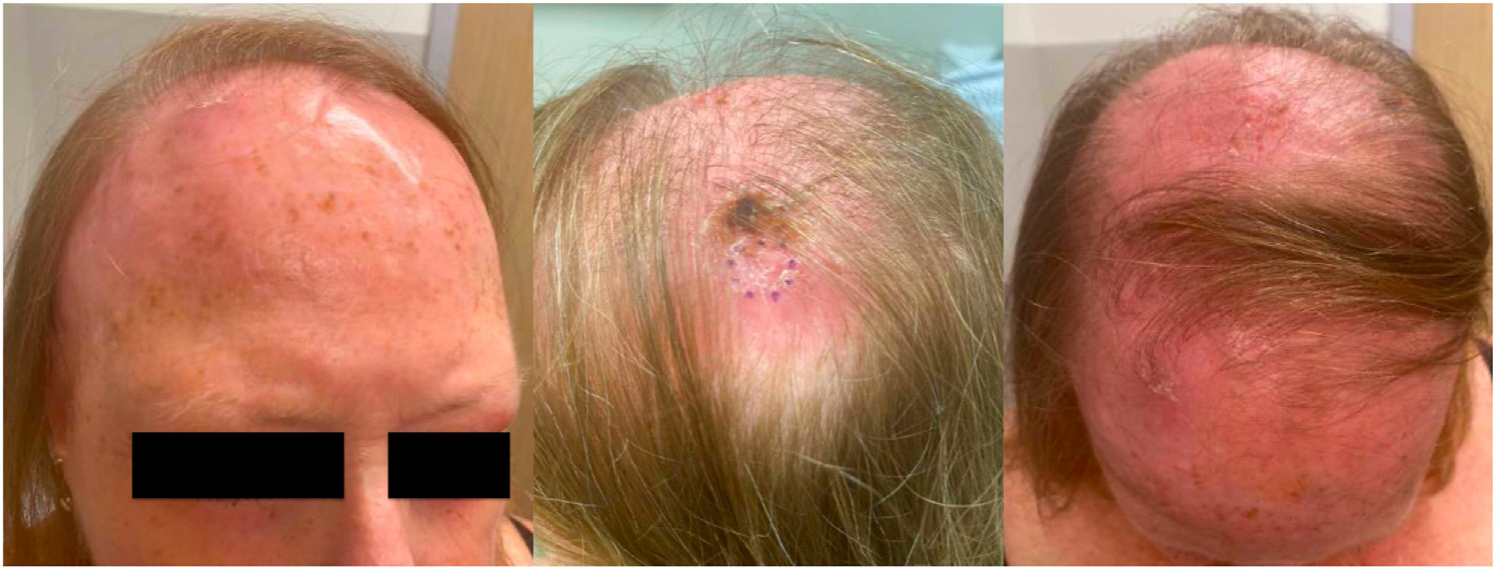
Cutaneous tumors of the scalp with diffuse thickening and erythema of the scalp.

A 70-year-old White woman with a past medical history notable for severe LPP treated with hydroxychloroquine for 10 years was referred to our dermatology and combined dermatology/oncology clinics for rapidly enlarging scalp nodules. At the time of evaluation, she had bilateral parietal and frontal scalp tumors (Fig 1) that evolved from a singular lesion 3 months prior. She likewise reported progressive hair loss and tenderness during this time. The tumors and associated symptoms were only minimally responsive to treatment with clobetasol and a concomitant prednisone taper.

**Fig 2:**
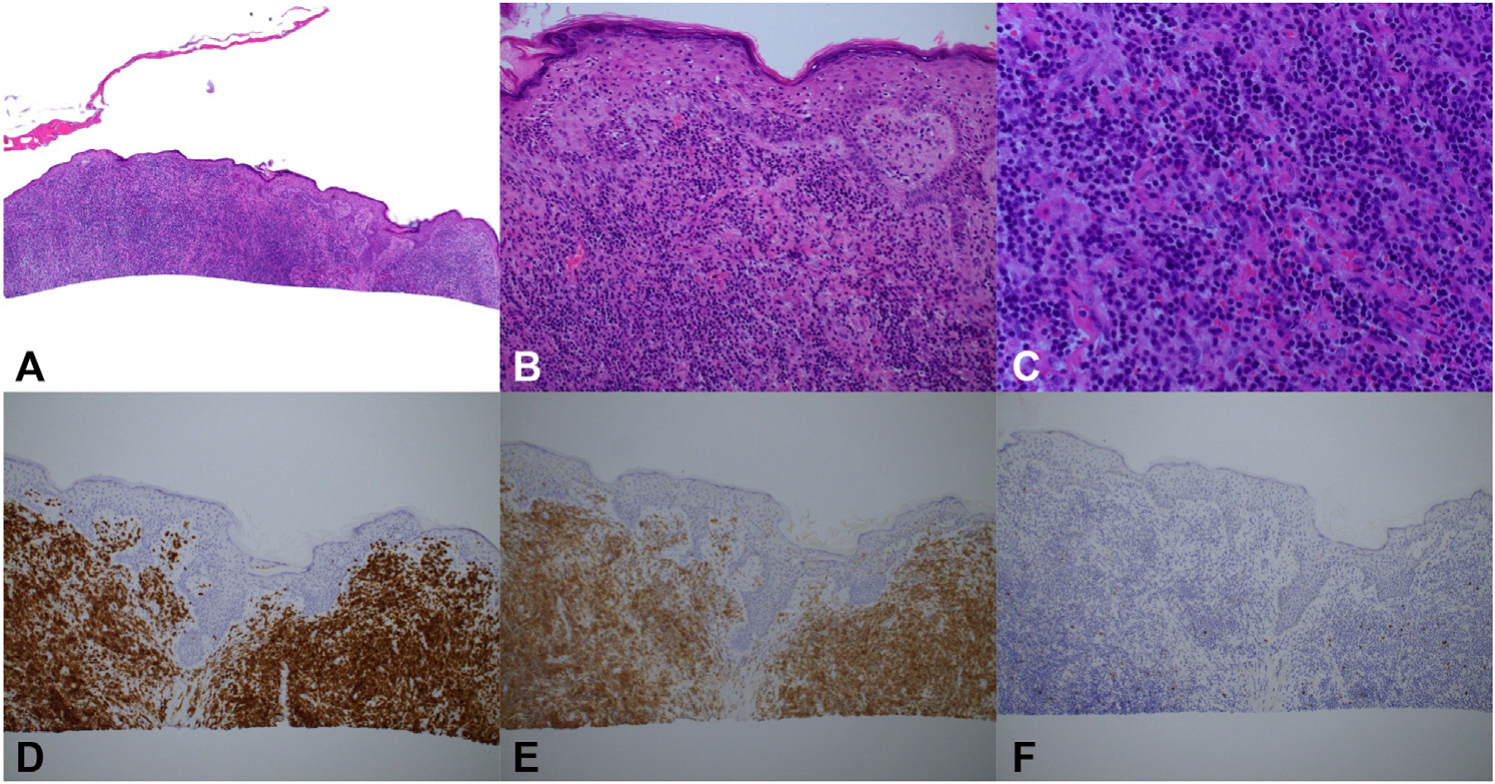
Representative histology images from shave biopsies of the rapidly enlarging scalp tumors of the vertex scalp shown in Fig 1. Shown are hematoxylin and eosin (H&E) stained slides at 40× (**A**), 200× (**B**), and 400× (**C**) magnification, respectively. A superficial and deep dermal subcutaneous infiltrate of lymphoid cells are appreciable with rare involvement at the dermal-epidermal junction and sparing of the hair follicles. CD3 (**D**), CD4 (**E**), and CD8 (**F**) stains are also shown and demonstrate strong CD3 and CD4 positivity of the lymphoid infiltrate with a CD4:CD8 ratio of approximately 20:1.

Biopsies of the bilateral vertex scalp revealed a striking superficial and deep dermal subcutaneous infiltrate of small to medium-sized lymphoid cells arranged in sheets. The lymphoid cells demonstrated minimal eosinophilic cytoplasm, regular nuclear contours, and inconspicuous nucleoli. Only rare lymphocytes were seen at the dermal-epidermal junction and the hair follicles were spared. The atypical lymphoid infiltrate was characterized by CD3, CD4, CD5, and TCRBetaF1 positivity, as well as CD30, CD10, CD56, CD123, and TCRDelta negativity. EBER in situ hybridization and PD1 were negative. The CD4:CD8 ratio was 20:1. T cell receptor beta and gamma gene rearrangements were also positive and showed the same clone at 2 separate sites (Fig 2). Of note, the tissue block and slides from the patient’s historical diagnostic LPP biopsy were no longer available for comparative analysis.

A complete workup was conducted to assess extracutaneous involvement and rule out systemic lymphoma. Laboratory workup was notable for a mild stable normocytic anemia, normal metabolic panel, and normal lactate dehydrogenase. Uric acid was mildy elevated at 7.1 mg/dL. Human T-cell lymphotropic virus type 1, HIV, and hepatitis panels were all negative. Peripheral blood flow cytometry demonstrated an aberrant CD4+ T-cell population which compromised 5.3% of the total population. PET-CT was notable for fluorodeoxyglucose avidity of the bilateral parietal scalp but showed no evidence of lymph node or other organ involvement. Bone marrow biopsy demonstrated a hypercellular core with maturing trilineage hematopoiesis and involvement of a peripheral T-cell lymphoma representing 50% to 60% of the marrow cellularity. Fluorescence *in-situ* hybridization analysis revealed a *MYC* gain-of-function mutation. Clinicopathologic correlation led to a diagnosis of MTCN.

**Fig 3:**
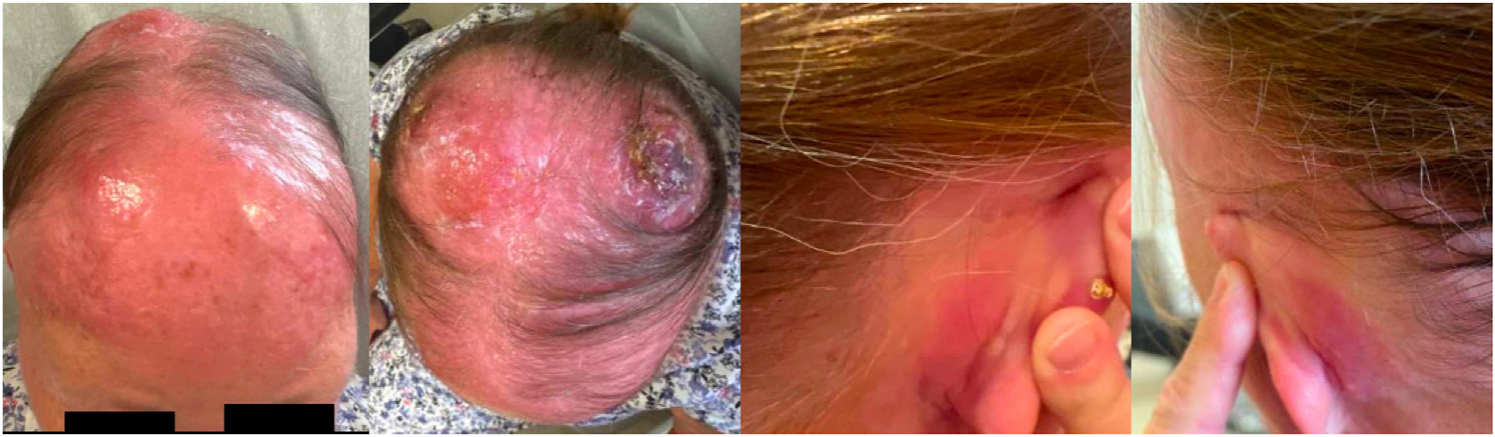
New tumor development with worsening erythema and enlarged pre-existing nodules at follow-up appointment 20 days from initial.

**Fig 4:**
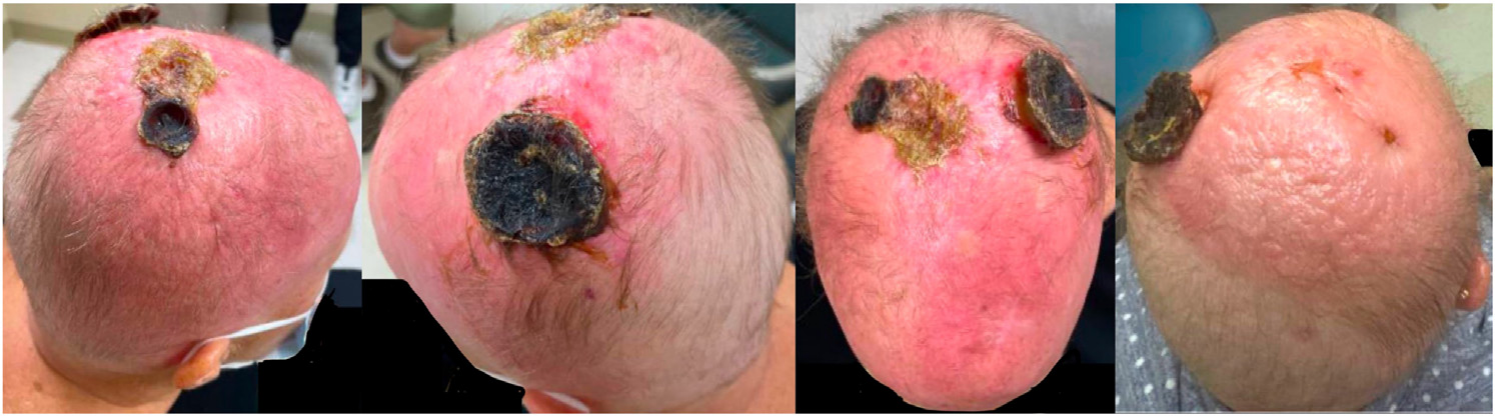
Image of lesional improvement following initiation of chemotherapy with CHOP.

The patient’s scalp lesions continued to rapidly worsen, and she developed diffuse thickening of the entire scalp (Fig 3) prompting urgent initiation of chemotherapy. She was started on cyclophosphamide, hydroxydanorubicin, oncovin, and prednisone (CHOP) chemotherapy regimen (cyclophosphamide, doxorubicin, vincristine, and prednisone) with a goal of 6 cycles total. Prophylactic care with granulocyte colony-stimulating factor (Neulasta) and valacyclovir was also initiated. The patient responded rapidly to CHOP therapy with significant decreases in tumor size and healing with black/brown crust after the first 2 treatment cycles (Fig 4). Of note, the patient’s medical course was complicated by eyelid pruritis and edema, neutropenic fever and port infection, axillary nodules, and septic shock secondary to *Pseudomonas* bacteremia due to ecthyma gangrenosum arising from infected scalp tumors. Following the multiple infections, she was started on prophylactic levofloxacin and her chemotherapy dose was temporarily reduced by 25%. The patient will continue with CHOP therapy and future consolidative autologous stem cell transplant is being considered.

## Discussion

To the best of our knowledge, there are no previously reported cases of concurrent MTCN and LPP. Based on the pathologic features, this lymphoma is best classified as PTCL-NOS. The current (2024) World Health Organization classification of hematolymphoid tumors recognizes 2 categories of PTCL-NOS: nodal and primary cutaneous.^5^ Primary cutaneous PTCL-NOS is rare and appears to have an aggressive clinical course similar to its nodal counterpart.^6^^,^^7^ The distribution of lymphoma in this patient, with involvement of the skin and bone marrow and lack of nodal disease, is unusual, and would not be typical of either nodal or primary cutaneous PTCL-NOS.

The striking accumulation of malignant lymphocytes localized at the site of the patient’s long-standing LPP is notable, and because of this feature we propose that this patient’s lymphoma is likely related to her previously diagnosed LPP. Many lymphoma types appear to be associated with concurrent inflammation. These include the following:

- Lymphomas associated with chronic infection. The most conclusively proved link between chronic infection and lymphoma is the association of gastric marginal zone lymphoma with *Helicobacter pylori* infection, although infection by *Chlamydia psittaci, Borrelia burgdorferi,* and *Campylobacter jejuni* have also been proposed as inciting agents in other lymphoma types.^8^
- Lymphomas such as classic Hodgkin lymphoma and anaplastic large cell lymphoma, in which the malignant cells are often present in an inflammatory cell antitumoral milieu.^9^
- Differing lymphoma types arising in patients with chronic inflammatory/autoimmune disorders such as rheumatoid arthritis and inflammatory bowel disease.^10^

Because of its aggressive clinical behavior, anthracycline-based chemotherapy regimens are the standard of care for PTCL-NOS.^7^ For better outcomes, most experts recommend adding etoposide (cyclophosphamide, hydroxydanorubicin, oncovin, etopside, and prednisone) for patients who are medically fit and younger than 60 years old. Our patient responded rapidly to CHOP, though complete clinical resolution of the patient’s tumors has not been achieved following 2 cycles. Many patients with PTCL-NOS may require autologous stem cell transplants, especially in refractory or relapsed disease.

## Conflicts of interest

None disclosed.
